# Length of biliopancreatic limb in Roux-en-Y gastric bypass and its impact on post-operative outcomes in metabolic and obesity surgery—systematic review and meta-analysis

**DOI:** 10.1038/s41366-022-01186-0

**Published:** 2022-08-04

**Authors:** Anna Kamocka, Swathikan Chidambaram, Simon Erridge, Gauri Vithlani, Alexander Dimitri Miras, Sanjay Purkayastha

**Affiliations:** 1 Department of Metabolism, Digestion and Reproduction, Hammersmith Hospital, Imperial College London, 6th Floor Commonwealth Building, Du Cane Road, London, W12 0NN UK; 2 Department of Surgery and Cancer, Imperial College London, London, SW7 2AZ UK; 3 School of Medicine, Ulster University, London, UK

**Keywords:** Translational research, Obesity

## Abstract

**Background:**

Roux-en-Y gastric bypass (RYGB) is a gold-standard procedure for treatment of obesity and associated comorbidities. No consensus on the optimal design of this operation has been achieved, with various lengths of bypassed small bowel limb lengths being used by bariatric surgeons. This aim of this systematic review and meta-analysis was to determine whether biliopancreatic limb (BPL) length in RYGB affects postoperative outcomes including superior reduction in weight, body mass index (BMI), and resolution of metabolic comorbidities associated with obesity.

**Methods:**

A systematic search of the literature was conducted up until 1st June 2021. Meta-analysis of primary outcomes was performed utilising a random-effects model. Statistical significance was determined by *p* value < 0.05.

**Results:**

Ten randomised controlled trials were included in the final quantitative analysis. No difference in outcomes following short versus long BLP in RYGB was identified at 12–72 months post-operatively, namely in BMI reduction, remission or improvement of type 2 diabetes mellitus, hypertension, dyslipidaemia, and complications (*p* > 0.05). Even though results of four studies showed superior total body weight loss in the long BPL cohorts at 24 months post-operatively (pooled mean difference −6.92, 95% CI –12.37, −1.48, *p* = 0.01), this outcome was not observed at any other timepoint.

**Conclusion:**

Based on the outcomes of the present study, there is no definitive evidence to suggest that alteration of the BPL affects the quantity of weight loss or resolution of co-existent metabolic comorbidities associated with obesity.

## Introduction

Obesity is a multi-system disease associated with the development of metabolic sequalae, such as type 2 diabetes mellitus (T2DM), hypertension, obstructive sleep apnoea and other obesity-associated comorbidities [1]. In 2015, excess weight contributed to 4 million global deaths and 120 million disability-adjusted life years [2]. Recent estimates suggest that ~5% of children and 12% of adults globally have obesity, with incidence increasing annually [2].

As supported by a large body of evidence, the most effective and durable treatment for obesity and associated comorbidities is bariatric surgery [3–14]. The first gastric bypass for weight loss was performed in 1966 by Mason and Ito [15]. Subsequently, studies have sought to optimise outcomes and minimise surgical risks by modifying gastric bypass. The first documented laparoscopic Roux-en-Y Gastric Bypass (RYGB) for treatment of obesity was performed by Wittgrove and Clarke in 1993 [16]. Almost three decades later, it remains the gold-standard metabolic procedure [17]. RYGB has been shown to be associated with 25–35% total body weight loss [18]. Moreover, a 40–75% incidence of T2DM remission is observed with a mean reduction in glycated haemoglobin of ~22 mmol/mol at 1–2 years post-operatively and reduced burden of diabetic medications or even a complete cessation of pharmacotherapy [19–21]. RYGB involves the formation of a small gastric pouch formed over the lesser curve of the stomach which is anastomosed to an alimentary (Roux) small bowel limb. The alimentary limb (AL) is then anastomosed to a biliopancreatic limb (BPL). A segment of the small bowel distal to the jejuno-jejunal anastomosis of the AL and BPL, the common channel, and its length varies depending on the total small bowel length of an individual.

Even though RYGB has been widely used as a weight loss and metabolic procedure, no consensus has been reached with regards to the optimal length of the bypassed small bowel segments. Significant variations in the total small bowel length between individuals (3–11 m) [22, 23] make defining widely applicable standards even more challenging. Furthermore, significant heterogeneity exists in the studies reviewing the lengths of the bypassed small bowel limbs, which makes it difficult to compare the results and draw clear conclusions [24]. It has been shown that increasing the length of the AL brings very little or no significant improvement in weight loss [25, 26] or long-term remission of metabolic syndrome-associated diseases [24].

Therefore, more attention has been brought to the length of the BP limb and the common channel. Several prospective studies have demonstrated promising results. Nergaard et al. compared a standard RYGB (150 cm AL with 60 cm BPL) to a long BPL RYGB (200 cm) with a short AL (60 cm) in 187 patients. Over 7 years follow up, increased long-term weight loss was shown in the long BPL group. However, no difference in the remission of obesity-related comorbidities was observed and more nutritional deficiencies were recorded in this group [27]. The authors speculated that the superiority of the 200 cm BPL in weight loss outcomes was because such a long bypass of proximal bowel would bypass most of the foregut, including all of the jejunum. Hence, the gastrointestinal anastomosis was a gastro-ileostomy, not a gastro-jejunostomy. Undigested nutrients entering ileum directly could have a more potent impact on nutrient sensing and eating behaviours and bypassing such a large proportion of foregut could have stimulated more potent enteroendocrine response and gut hormone secretion [28]. Nora et al. led a prospective study of 94 patients with obesity and T2DM who underwent RYGB with a 200 cm BPL and a 120 cm AL [29]. The cohort of 40 (43%) patients that completed the 3-year follow up lost 25% body weight, stopped all their glucose-lowering medications, and reduced their HbA1c% by 0.9% from a baseline of 6.7%, achieving 100% T2DM remission rate. Therefore, this study showed that a longer BPL may be associated with superior outcomes with respect to glycaemic control compared to a standard RYGB, which made it more comparable to the biliopancreatic diversion (BPD). However, it was a prospective observational study with almost 60% of patients lost to follow up at 3 years, hence reporting bias is possible. Similarly, in a retrospective analysis of 671 patients with an average BMI of 50 kg/m^2^ and 10 year follow up, Shah et al. concluded that 200 cm BPL provides superior outcomes in terms of excess weight loss (EWL), less weight regain, and remission of comorbidities. They argued that greater weight loss is achieved with shortening of the total alimentary channel (i.e. alimentary limb and common limb) and advise BPL of 200 cm and AL of 100 cm in order to achieve optimal outcomes [30]. A systematic review by Zorrilla-Nunez et al. of 13 predominantly prospective studies as well as several RCTs suggested that length of the BPL may affect post-operative outcomes after RYGB, with superior weight loss associated with a longer BPL length [31]. With several observational studies and some RCTs supporting or disputing the importance of BPL elongation in RYGB, no definite conclusion has been reached to date.

## Aim

The aim of this systematic review and meta-analysis was therefore to determine whether BPL length in RYGB affects postoperative outcomes including change in weight, body mass index (BMI), and resolution of metabolic comorbidities associated with obesity.

## Methods

### Search strategy and selection criteria for studies

**Fig. 1:**
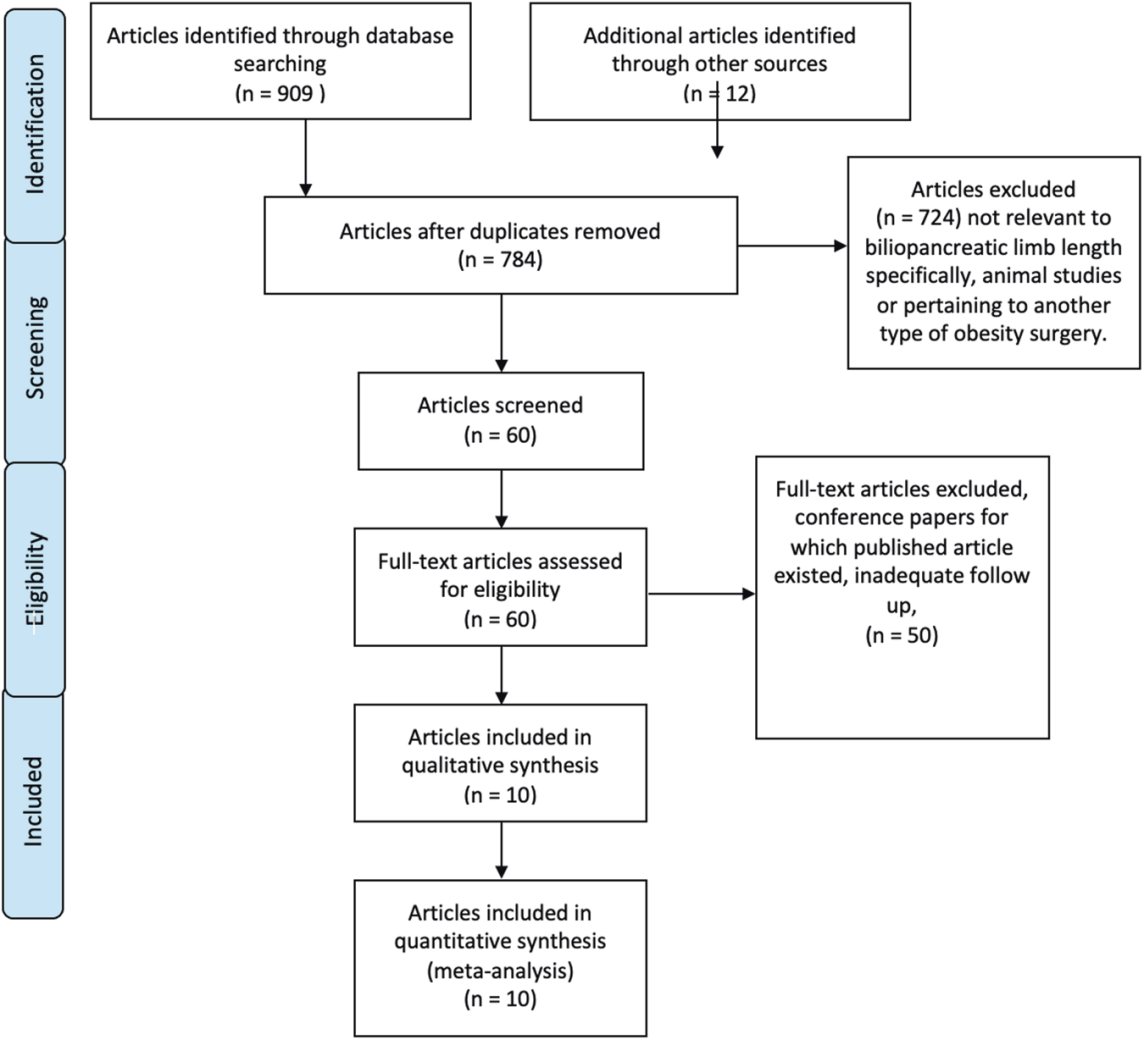
PRISMA diagram.

A systematic search was conducted in accordance with PRISMA guidelines (Fig. 1) of articles published in MEDLINE, EMBASE, and CENTRAL databases until 1st June 2021. Search terms included both keywords and MeSH terms. Full search strategy is detailed in supplementary Appendix 1. Reference lists of included studies and previous reviews were hand-searched to identify further studies of interest. Search results were limited to English language. The search was performed independently by three authors (S.C., S.E., G.V). Studies were reviewed independently for inclusion in full-text review, with any agreement to be resolved by the senior author (S.P.) if applicable, however, this was not required in this case. The following inclusion and exclusion criteria were utilised.

Inclusion criteria:

1. Randomised control trial.
2. Minimum of a 1 year follow up.
3. Trial compared two or more different lengths of BPL in RYGB.
4. Reported quantitative outcome data, such as BMI loss, percentage EWL, postoperative BMI and/or obesity-related co-morbidities
5. Studies in the English language.

Exclusion Criteria:

1. Compared RYGB and other types of bariatric surgeries such as gastric banding and sleeve gastrectomy.
2. Participants underwent other surgical or medical treatments.
3. Animal studies.
4. Studies where full text could not be obtained, despite contacting corresponding author of study.

### Quality of evidence and bias risk assessment

Quality of evidence for each study was evaluated in line with the GRADE framework [32]. Furthermore, the Cochrane risk of bias tool for randomised trials was used to assess the risk of bias for each study, including bias arising from randomisation and allocation sequence concealment (selection bias), performance bias, attrition bias, detection bias, reporting bias and other potential sources of bias [32].

### Data extraction

Participant characteristics were collected including age, sex, baseline weight, baseline BMI, comorbidities, and ethnicity. Intraoperative data collected included: size of gastric pouch, length of BPL, length of AL, and length of common channel where measured. Finally, post-operative outcomes collected included changes in percentage excess weight, percentage excess BMI, HbA1c, blood pressure and lipid parameters. Rate of postoperative adverse events was also extracted.

### Meta-analysis

#### Measures of treatment effect

The pooled mean difference (MD) and its 95% confidence intervals (CIs) were calculated for continuous outcomes. Where studies had continuous variables that used different scales/instruments, we calculated standardised mean differences (SMD) with 95% CI. For studies that reported baseline and endpoint data, we calculated the standard deviation of the mean change from the baseline according to reported CI.

#### Unit of analysis issues

Unit of analysis issues was dealt with according to specific study design. The relevance of each intervention group to this systematic review was determined by what was previously set out in the selection criteria based on the types of population and types of intervention. The control arm was divided equally by the number of included intervention groups in studies that contain two or more groups. If the study already presented separate subgroup analyses, then the control group was considered as a whole.

#### Dealing with missing data

Where necessary, the authors of selected studies were contacted to obtain any missing data. When this was not possible, standard deviations or correlation coefficients were calculated using the data available. If data continued to be unavailable, we conducted an available case analysis by excluding unavailable data points.

#### Assessment of heterogeneity

Clinical heterogeneity (differences in participant type or characteristics, timing of outcome measurements and intervention characteristics) was assessed by firstly reviewing the treatments used across studies, in addition to clinicopathologic (characteristics of included participants to assess for any substantial differences. Statistical heterogeneity was assessed using the 𝛘^2^ test and I^2^ statistic. A *p* value of 0.05 was considered statistically significant for the 𝛘^2^ test. The *I*^2^ statistic was used to quantify the proportion of variation between studies that is due to heterogeneity rather than to chance. This interpretation was in keeping with the Cochrane Handbook of systematic reviews [32]. An *I*^2^ value of 0–40% indicates heterogeneity may not be important; 30–60% indicates moderate heterogeneity; 50–90% indicates substantial heterogeneity and 75–100% indicates considerable heterogeneity. Forest plots were created and visibly inspected to identify any outliers. A sensitivity analysis was conducted if any outliers are found to explore the potential explanations for the observed heterogeneity.

#### Assessment of reporting biases

Reporting bias was assessed by comparing pre-specified outcomes in pre-trial registry entries/study protocols (where available) to outcomes reported in final manuscripts. If registry entries or protocols were unavailable, reporting bias was assessed by comparing outcomes specified in the methodology compared to those reported in the results section. Funnel plots were not created, due to fewer than ten studies being included in the final analysis limiting their utility as previously outlined [33].

#### Data synthesis

For continuous outcomes, a pooled MD and 95% CI were calculated. However, in studies using different scales the SMD and 95% CI were calculated. Odds ratios with 95% CI were also calculated for data presented as frequencies. A decision was made not to pool studies together if considerable clinical heterogeneity existed. A random-effects model was used to pool data, instead of a fixed-effects model, if clinical heterogeneity was acceptable and the data presented in the literature was substantially heterogenous in nature. Statistical significance was set at *p* value < 0.05. All data were analysed using Review Manager v5.3.

## Results

### Study selection

The database search yielded a total of 909 studies, and an additional 12 studies were identified through other sources. Of these, 137 duplicates were removed. Titles and abstracts of the remaining 784 studies were assessed for eligibility. Records were excluded if they were not relevant to biliopancreatic limb length specifically, were animal studies or pertained to another type of obesity surgery (*n* = 724). Further 50 studies were excluded after full-text review due to incompatible outcome measures or study design. Ten studies were included in the final data synthesis (Fig. 1). A summary of included studies is presented in Table 1.

**Table 1:** Randomised controlled trials included in systematic review.

Authors	Year	Setting	Participants (n)	Follow up	Preoperative BMI Short Limb (kg/m^2^)	Preoperative BMI Long Limb (kg/m^2^)	Biliopancreatic Limb Length (short) (cm)	Alimentary Limb Length (short) (cm)	Biliopancreatic Limb Length (long) (cm)	Alimentary Limb Length (long) (cm)	Reported outcomes
Brolin et al.	1992	United States	45	12–86 months	63.4 ± 10.0 (48.8 - 94.6).	61.6 ± 9.0 (51.8 – 89.9)	15	75	30	150	Weight loss, BMI change, Complications, Mortality
Inabnet et al.	2005	United States	48	24 months	44.6 ± 3.0	44.9 ± 2.9	50	100	100	150	Weight loss, BMI change, Complications
Pinheiro et al.	2007	Brazil	105	Up to 48 months	53.4 ± NR	54.7 ± NR	50	150	150	250	Weight loss, T2DM improvement, HTN improvement, Dyslipidaemia improvement
Nergaard et al.	2014	Iceland/Norway/Sweden	187	Up to 96 months	43.7 (38–68)	44.5 (39–70)	60	150	200	60	BMI change, T2DM improvement, HTN improvement, Complications
Valezi et al.^a^	2014	Brazil	120	12 months	46.9 ± 5.69 (100 cm) / 47.64 ± 5.72 (150 cm)	46.81 ± 5.50 (100 cm) / 46.10 ± 5.39 (150 cm)	50	100 / 150^a^	100	100 / 150^a^	Weight loss
Homan et al.	2018	Netherlands	146	48 months	45 ± 5	43 ± 5	75	150	150	75	Weight loss, BMI change, T2DM improvement, HTN improvement, Dyslipidaemia improvement, Complications, Mortality
Boerboom et al.	2019	Netherlands	146	48 months	42 ± 4	43 ± 5	75	150	150	75	Weight loss, BMI change, T2DM improvement, HTN improvement, Dyslipidaemia improvement, Complications, Mortality
Ruiz Tovar et al.	2019	Spain	506	5 years	44.2 ± 5.2	44.1 ± 4.1	70	150	120	150	BMI change, T2DM improvement, HTN improvement, Dyslipidaemia improvement,
Miras et al.	2020	United Kingdom	53	12 months	42 ± 6	43 ± 8	50	100	150	100	Weight loss, HTN improvement, Complications
Nergård et al.	2020	Sweden	140	5 years	55.8 ± 6.0	55.6 ± 6.0	60	150	200	Variable (common channel 150 cm)	Weight loss, BMI change, T2DM improvement,

*BMI* body mass index, *HTN* hypertension, *NR* not reported, *T2DM* type 2 diabetes mellitus.

^a^In the study by Valezi et al. there were four study arms comparing both biliopancreatic and alimentary limb lengths.

### Design of RYGB

In the ten included RCTs, short (or standard) BPL length varied from 15 to 75 cm, with 50 cm being the most used. In the long BPL cohort, BPL length ranged between 30 and 200 cm, with 150 cm being bypassed most frequently. Reported alimentary limb length was 60–250 cm, with 150 cm being the most common measurement. Seven trials compared short vs long BPL whilst forming an AL of a varying length, whereas remaining three RCTs set up a single standard AL length across study arms.

### Total body weight loss at 12 months

**Fig. 2:**
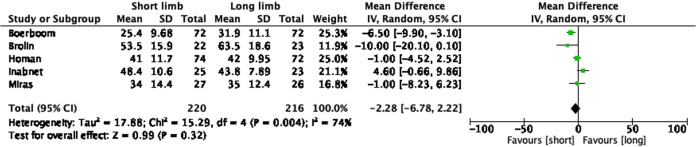
Forest plot of studies assessing total body weight loss (kg) at 12 months.

Five studies included percentage of the total body weight loss records at 12 months as the primary outcome measure (Fig. 2). The total sample size was 436 patients with similar numbers in the short limb (*n* = 220) and long limb (*n* = 216) cohorts. The random-effects model demonstrated no statistically significant difference between the two cohorts (pooled mean difference −2.28, 95% CI −6.78, 2.22, *p* = 0.32).

### Medium and long-term total body weight loss

**Fig. 3:**
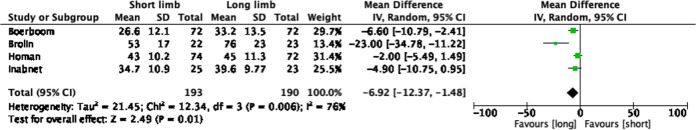
Forest plot of studies assessing total body weight loss (kg) at 24 months.

Four studies evaluated percentage of the total body weight loss at 24 months (Fig. 3). The total sample size was 383 patients with similar numbers in the short limb (*n* = 193) and long limb (*n* = 190) cohorts. The random-effects model demonstrated a higher weight loss of statistical significance for the long limb cohort (pooled mean difference −6.92, 95% CI –12.37, −1.48, *p* = 0.01).

**Fig. 4:**
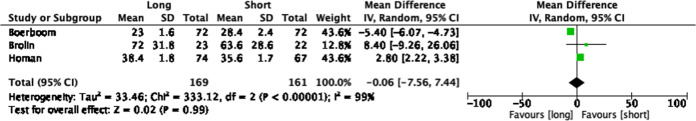
Forest plot of studies assessing total body weight loss (kg) at 48–72 months.

However, superior weight loss was not observed in the longer term follow up (Fig. 4). Of three studies with total sample size of 330 patients (short limb *n* = 161, long limb *n* = 169), providing data for total body weight loss at a follow-up period at 48–72 months, the random-effects model demonstrated no statistically significant difference between the two cohorts (pooled mean difference −0.06, 95% CI –7.56, −7.44, *p* = 0.99).

### Reduction in BMI

**Fig. 5:**
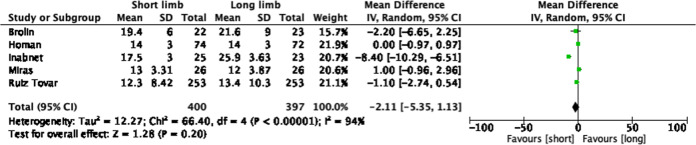
Forest plot of studies assessing reduction in BMI (kg/m^2^) at 12 months.

Five studies included measured change in body mass index at 12 months as a primary outcome measure (Fig. 5). The total sample size was 797 patients with similar numbers in the short limb (*n* = 400) and long limb (*n* = 397) cohorts. The random-effects model demonstrated no statistically significant difference between the two cohorts (pooled mean difference −2.11, 95% CI −5.35, 1.13, *p* = 0.20).

**Fig. 6:**
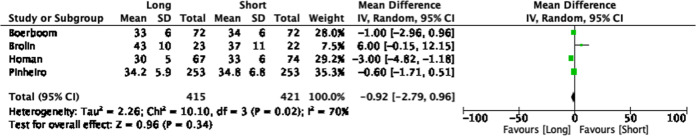
Forest plot of studies assessing reduction in BMI (kg/m^2^) at 48–72 months.

These outcomes were sustained in a longer follow up (Fig. 6). In four studies with total sample size of 836 patients (short limb *n* = 421, long limb *n* = 415), the random-effects model demonstrated no statistically significant difference between the two cohorts (pooled mean difference −0.92, 95% CI –2.79,0.96, *p* = 0.34) at 48–72 months post-operatively.

The proportion of patients who were reported to have metabolic resolution of their comorbidities or experience an adverse event is recorded in Table 2.

**Table 2:** Reported metabolic outcomes and complications.

Authors	Participants (*n*)		Remission/improvement in type 2 diabetes mellitus^a^ (%)		Remission/improvement in hypertension (%)		Remission/improvement in dyslipidaemia (%)		Complication Incidence (%)	
	Short Limb	Long Limb	Short Limb	Long Limb	Short Limb	Long Limb	Short Limb	Long Limb	Short Limb	Long Limb
Brolin et al.	22	23	NR	NR	NR	NR	NR	NR	NR	NR
Inabnet et al.	25	23	NR	NR	NR	NR	NR	NR	13/25 (52.0%)	13/23 (56.5%)
Pinheiro et al.	57	48	54/55 (98.2%)	43/45 (95.6%)	13/26 (50.0%)	13/21 (63.0%)	30/52 (57.7%)	29/41 (70.7%)	NR	NR
Nergaard et al.	93	94	12/17 (70.6%)	17/22 (77.3%)	22/31 (71.0%)	17/28 (60.7%)	NR	NR	NR	NR
Valezi et al.	49	45	NR	NR	NR	NR	NR	NR	NR	NR
Homan et al.	74	72	21/22 (95.5%)	22/22 (100.0%)	19/24 (79.2%)	26/33 (78.8%)	50/60 (83.3%)	53/58 (91.4%)	29/74 (39.2%)	27/72 (37.5%)
Boerboom et al.	72	72	11/15 (73.3%)	6/9 (66.7%)	9/18 (50.0%)	10/24 (37.5%)	8/13 (61.5%)	4/7 (57.1%)	28/72 (38.9%)	29/72 (40.3%)
Ruiz Tovar et al.	253	253	30/90 (33.3%)	30/85 (35.3%)	40/120 (33.3%)	42/112 (37.5%)	39/82 (47.6%)	41/80 (51.2%)	NR	NR
Miras et al.	27	26	16/26 (61.5%)	20/26 (76.9%)	NR	NR	NR	NR	18/27 (66.7%)	23/26 (88.5%)
Nergård et al.	74	66	13/15 (86.7%)	15/15 (100.0%)	12/23 (52.2%)	14/19 (73.7%)	NR	NR	7/74 (9.46%)	12/66 (18.2%)
Overall^b^	746	722	73.3%	77.3%	51.1%	60.7%	59.6%	63.9%	39.2%	40.3%

*NR* not reported.

^a^At longest reported follow-up.

^b^Overall values are presented as medians.

### Remission or improvement in T2DM

Three studies included measured remission or improvement in T2DM at 12 months as a secondary outcome measure (Supplementary Fig. 1). The total sample size was 334 patients with similar numbers in the short limb (*n* = 174) and long limb (*n* = 160) cohorts. The fixed-effects model demonstrated no statistically significant difference between the two cohorts (odds ratio 1.19, 95% CI 0.69, 2.04, *p* = 0.54).

These findings were sustained in five studies providing data on the long term follow up (Supplementary Fig. 2). The fixed-effects model in 373 patients (short limb *n* = 197, long limb *n* = 176) demonstrated no statistically significant difference between the two cohorts (odds ratio 1.11, 95% CI 0.64, 1.91, *p* = 0.71) in remission or improvement in T2DM at 24–60 months post-operatively.

### Remission or improvement in Hypertension

Five studies with total sample size of 420 patients (short limb *n* = 211, long limb *n* = 209) investigated remission or improvement in hypertension at a follow-up period ranging from 24–60 months (Supplementary Fig. 3). The fixed-effects model demonstrated no statistically significant difference between the two cohorts (odds ratio 1.24, 95% CI 0.83, 1.86, *p* = 0.29).

### Remission or improvement in dyslipidaemia

Four studies (399 patients, short limb *n* = 207, long limb *n* = 186) measured remission or improvement in dyslipidaemia at a follow-up period of 24–60 months (Supplementary Fig. 4). The fixed-effects model demonstrated no statistically significant difference between the two cohorts (odds ratio 1.40, 95% CI 0.90, 2.18, *p* = 0.14).

### Incidence of complications

Five studies recorded the incidence of post-operative complications as a secondary outcome measure (Supplementary Fig. 5). The total sample size was 531 patients with similar numbers in the short limb (*n* = 272) and long limb (*n* = 259) cohorts The fixed-effects model demonstrated no statistically significant difference between the two cohorts (odds ratio 1.27, 95% CI 0.87, 1.85, *p* = 0.22).

## Discussion

This systematic review and meta-analysis identified 10 RCTs comparing weight and metabolic outcomes after RYGB with long and short biliopancreatic limbs. Meta-analysis of clinical outcomes of these studies does not support the proposed theory on superior weight loss, improved glycaemic control nor higher remission of obesity-related comorbidities in RYGB with a long biliopancreatic limb. Even though there was superior weight loss of almost 7% in the long limb cohorts at 24 months after the surgery, it was an isolated finding not observed in the longer term follow up, hence does not seem to be clinically relevant. Moreover, the studies included in analysis of BMI and weight loss are similar suggesting the difference in total weight loss at 24 months is likely attributed to differences in baseline body composition, rather than secondary to different BP lengths. All but one study concentrated on reporting clinical outcomes. Miras et al [34] conducted a mechanistic study investigating impact of the length of intestinal bypass in RYGB on GLP-1 and glucose homoeostasis, including insulin secretion and sensitivity. Findings of this study also disputed the benefit of elongating BP limb on a physiological level, with no evidence on beneficial impact of elongating BP limb on fasting and post-prandial gut hormones secretion and glucose homoeostasis over a standard RYGB.

The theory, that the bypass of proximal small bowel has superior and weight loss-independent effects on glucose metabolism compared to the bariatric procedures that do not include an intestinal bypass, is based on outcomes of bariatric procedures such as BPD and RYGB having greater clinical effects on glucose control compared to the gastric band and sleeve gastrectomy. This has been demonstrated by clinical and mechanistic studies comparing RYGB to a gastric band and sleeve gastrectomy in both early and late post-operative stages [35–38]. Furthermore, early studies on an isolated bypass of the distal duodenum and proximal jejunum with endoscopic liner EndoBarrier^®^ demonstrated its metabolic impact on weight loss and glycaemic control. Whilst it causes only a small to moderate weight loss (8–16%) at 6–12 months [39, 40], it results in absolute reductions in HbA1c% of 1.2–2.4% (starting HbA1c 7.3–9.1%) in the same period of time [41–43].

BPD has been shown to lead to superior rates of T2DM remission when compared to RYGB with up to 95% patients fulfilling the criteria at 2 years, with an absolute reduction in HbA1c% of 3.9% [19]. Its use, however, is limited due to significant long-term nutritional complications [18]. The main difference between the RYGB and BPD is a much longer biliopancreatic limb and a shorter common channel in the latter. Therefore, multiple bariatric centres have attempted to modify alimentary and biliopancreatic limb lengths in the RYGB to optimise its outcomes.

Altering BP limb length can influence glucose homoeostasis and weight loss through several mechanisms. RYGB causes a large release of gut hormones such as GLP-1, oxyntomodulin and peptide YY after eating, leading to reductions in appetite and/or increases in insulin secretion [29, 44–49]. A longer BP limb in RYGB should enable faster delivery of undigested nutrients to the distal jejunum, where a greater number of gut endocrine L cells are present [50]. Therefore, it is expected that it will cause a greater release of gut hormones that will subsequently drive a higher secretion of postprandial insulin compared to the standard RYGB. Moreover, through bypassing a longer segment of the small bowel, the long-BP limb RYGB is expected to result in even higher than in the standard RYGB levels of circulating bile acids, gut microbiota and their metabolites and therefore even more potent effects on T2DM. Long-BP limb RYGB is also expected to increase hepatic and peripheral insulin sensitivity in a similar fashion as the BPD. At the same time, it is not expected to cause the side effects which are the limiting factor in the BPD use.

Even though no difference in complication rate was noted between RYGB with short versus long BPL, it does not seem justified to elongate most frequently used length of 50–60 cm with no evidence of it being beneficial. Findings of this systematic review and meta-analysis suggest that there is no benefit in elongating BPL beyond standard design of 75 cm or less. It may be since RYGB has already achieved its optimal outcomes with those shorter BPL limb lengths and perhaps more research into alimentary and common channel lengths optimisation would help in improving patients’ outcomes following RYGB. It is evident, however, that there is a paucity of research on the underlying mechanisms of metabolic disease resolution following surgery, with majority of studies concentrating on short to medium-term clinical outcomes only.

Difficulties in interpreting these studies lie in their heterogenous design, with various definitions of length of the short and long BP limbs. Furthermore, measuring total small bowel length and interpreting ratios of bypassed limbs, which may be of benefit with already known wide range of total small bowel length in humans, has not been a common practice. Moreover, additional heterogeneity was introduced through differences in length of follow up, basal BMI, as well as the proportion of female and male participants. Further shortcomings were noted in outcomes reporting. No widely accepted and standardised definitions for obesity-related comorbidities’ remission were used. There was additionally, no description on how diagnosis of remission or improvement was made, and those were commonly interpreted at a given study’s investigators discretion. Hence reported improvement or remission of T2DM, hypertension, and hypercholesterolaemia in those ten RCTs may have been based on different criteria. Finally, the length of follow up in most studies means that the medium to long-term outcomes of differences in BP limb length are even less well understood.

## Conclusions

This meta-analysis of RCTs assessing the difference in outcomes between short and long-length BPL in the setting of RYGB found no significant difference in weight change, resolution of metabolic comorbidities, or complications. Confounding factors include a significant degree of heterogeneity in the design of the studies, with varying biliopancreatic and alimentary limb lengths. Moreover, there remains a paucity of investigations into the physiological changes which result in the observed outcomes following RYGB. Through dedicated investigation this would allow a better understanding of mechanisms of action, thereby informing surgical design based upon a first-principles approach.

## Supplementary information

Supplementary Figure 1

Supplementary Figure 2

Supplementary Figure 3

Supplementary Figure 4

Supplementary Figure 5

Search Criteria Record

Supplementary Figure Legends

## Data Availability

The data used in this publication are readily available from original source papers included in the systematic review.
