# Supplementary figures and images for: Length of biliopancreatic limb in Roux-en-Y gastric bypass and its impact on post-operative outcomes in metabolic and obesity surgery—systematic review and meta-analysis
Source: Int J Obes (Lond). 2022 Aug 4;46(11):1983–91. doi: 10.1038/s41366-022-01186-0 (PMC9584808; doi:10.1038/s41366-022-01186-0)

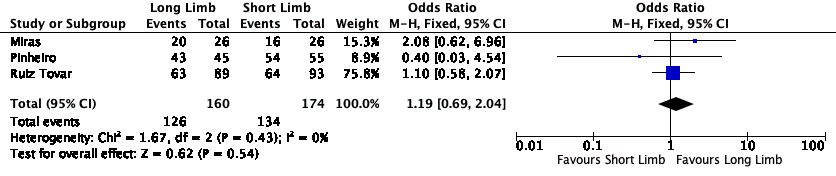

Supplement: Supplementary file 1 — Supplementary Figure 1 [file 41366_2022_1186_MOESM1_ESM.png]

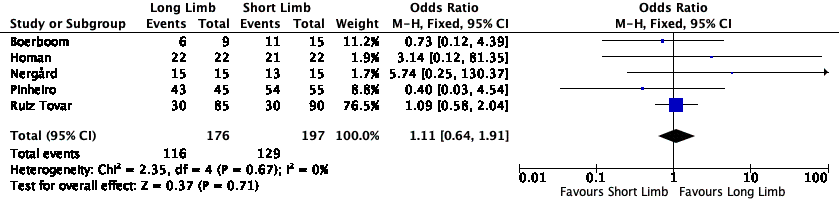

Supplement: Supplementary file 2 — Supplementary Figure 2 [file 41366_2022_1186_MOESM2_ESM.png]

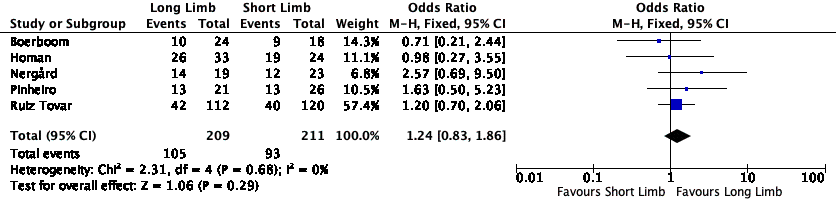

Supplement: Supplementary file 3 — Supplementary Figure 3 [file 41366_2022_1186_MOESM3_ESM.png]

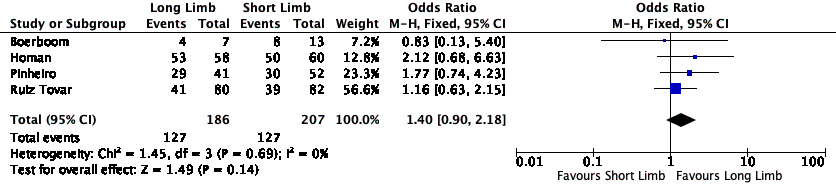

Supplement: Supplementary file 4 — Supplementary Figure 4 [file 41366_2022_1186_MOESM4_ESM.png]

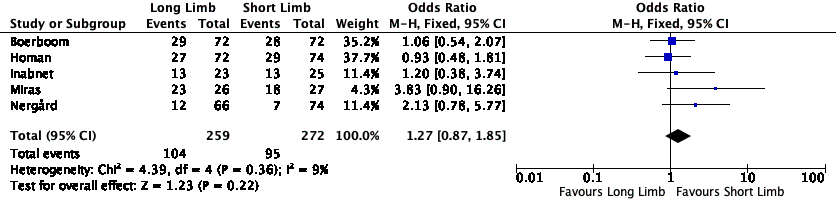

Supplement: Supplementary file 5 — Supplementary Figure 5 [file 41366_2022_1186_MOESM5_ESM.png]
