# Supplementary material for: Length of biliopancreatic limb in Roux-en-Y gastric bypass and its impact on post-operative outcomes in metabolic and obesity surgery—systematic review and meta-analysis
Source: Int J Obes (Lond). 2022 Aug 4;46(11):1983–91. doi: 10.1038/s41366-022-01186-0 (PMC9584808; doi:10.1038/s41366-022-01186-0)
Supplement: Supplementary file 6 — Search Criteria Record [file 41366_2022_1186_MOESM6_ESM.docx]

**Additional file 1**

**Search strategies and results**

**Table S1: Summary of Databases Searched**

| **Table** | **Vendor/ Interface** | **Database** | **Date searched** | **Searcher(s)** |
| --- | --- | --- | --- | --- |
| 1a | Ovid | Medline^®^ | 01/06/2021 | Swathikan Chidambaram, Simon Erridge, Gauri Vithlani |
| 1b | National Library of Medicine | PubMed | 01/06/2021 | Swathikan Chidambaram, Simon Erridge, Gauri Vithlani |
| 1c | Ovid | EMBASE^®^ | 01/06/2021 | Swathikan Chidambaram, Simon Erridge, Gauri Vithlani |
| 1d | Cochrane | CENTRAL | 01/06/2021 | Swathikan Chidambaram, Simon Erridge, Gauri Vithlani |

**Table S1a: Ovid Medline^®^ search strategy**

| **Provider/Interface** | Ovid |
| --- | --- |
| **Database** | Medline^®^ |
| **Date searched** | 01/06/2021 |
| **Search developer(s)** | Swathikan Chidambaram, Simon Erridge, Gauri Vithlani |
| **Limit to English** | Yes |

| 1 | exp Gastric Bypass/ |
| --- | --- |
| 2 | metabolic surgery.mp. |
| 3 | exp Bariatric Surgery/ |
| 4 | obesity surgery.mp. |
| 5 | weight loss surgery.mp. |
| 6 | exp Weight Loss/ |
| 7 | exp Metabolic Syndrome/ or exp Diabetes Mellitus |
| 8 | exp Obesity, Morbid/ or exp Obesity/ or exp Obesity Management |
| 9 | surgery.mp. or exp General Surgery/ |
| 10 | surgical management.mp. |
| 11 | 9 or 10 |
| 12 | 6 and 11 |
| 13 | 7 and 11 |
| 14 | 8 and 11 |
| 15 | 1 or 2 or 3 or 3 or 5 or 12 or 13 or 14 |
| 16 | pancreatobiliary.mp. |
| 17 | exp Biliopancreatic Diversion/ or biliopancreatic.mp. |
| 18 | exp Intestine, Small/ or small bowel.mp. |
| 19 | 16 or 17 or 18 |
| 20 | limb length.mp. |
| 21 | long limb.mp |
| 22 | long tract.mp |
| 23 | length.mp |
| 24 | surgical technique.mp |
| 25 | technique.mp |
| 26 | operative technique.mp. |
| 27 | 20 or 21 or 22 or 23 or 24 or 25 or 26 |
| 28 | randomized control trial .mp. |
| 29 | controlled clinical trial.mp. or exp Controlled Clinical Trial/ |
| 30 | exp Randomized Contolled Trial/ or randomized.mp |
| 31 | exp Clinical Trial/ or trial.mp |
| 32 | 28 or 29 or 30 or 31 |
| 33 | 15 and 27 and 32 |

**S1b: PubMed EMBASE**^®^ **search strategy**

| **Provider/Interface** | PubMed |  |
| --- | --- | --- |
| **Database** | Embase® | |
| **Date searched** | 01/06/2021 | |
| **Search developer(s)** | Swathikan Chidambaram, Simon Erridge, Gauri Vithlani | |
| **Limit to English** | Yes | |

| 1 | Gastric Bypass |
| --- | --- |
| 2 | metabolic surgery |
| 3 | Bariatric Surgery |
| 4 | obesity surgery |
| 5 | weight loss surgery |
| 6 | Weight Loss |
| 7 | Metabolic Syndrome OR Diabetes Mellitus |
| 8 | Obesity |
| 9 | Surgery |
| 10 | Surgical Management |
| 11 | 9 OR 10 |
| 12 | 6 AND 11 |
| 13 | 7 AND 11 |
| 14 | 8 AND 11 |
| 15 | 1 OR 2 OR 3 OR 3 OR 5 OR 12 OR 13 OR 14 |
| 16 | pancreatobiliary |
| 17 | Biliopancreatic Diversion OR biliopancreatic |
| 18 | small intestine OR small bowel |
| 19 | 16 OR 17 OR 18 |
| 20 | limb length |
| 21 | long limb |
| 22 | long tract |
| 23 | length |
| 24 | surgical technique |
| 25 | technique |
| 26 | operative technique |
| 27 | 20 OR 21 OR 22 OR 23 OR 24 OR 25 OR 26 |
| 28 | randomized control trial OR randomised control trial |
| 29 | controlled clinical trial |
| 30 | randomized OR randomised |
| 31 | clinical trial |
| 32 | 28 OR 29 OR 30 OR 31 |
| 33 | 15 AND 27 AND 32 |

**S1c: Ovid EMBASE**^®^ **search strategy**

| **Provider/Interface** | Ovid |  |
| --- | --- | --- |
| **Database** | Embase® | |
| **Date searched** | 01/06/2021 | |
| **Search developer(s)** | Swathikan Chidambaram, Simon Erridge, Gauri Vithlani | |
| **Limit to English** | Yes | |

| 1 | exp Gastric Bypass/ |
| --- | --- |
| 2 | metabolic surgery.mp. |
| 3 | exp Bariatric Surgery/ |
| 4 | obesity surgery.mp. |
| 5 | weight loss surgery.mp. |
| 6 | exp Weight Loss/ |
| 7 | exp Metabolic Syndrome/ or exp Diabetes Mellitus |
| 8 | exp Obesity, Morbid/ or exp Obesity/ or exp Obesity Management |
| 9 | surgery.mp. or exp General Surgery/ |
| 10 | surgical management.mp. |
| 11 | 9 or 10 |
| 12 | 6 and 11 |
| 13 | 7 and 11 |
| 14 | 8 and 11 |
| 15 | 1 or 2 or 3 or 3 or 5 or 12 or 13 or 14 |
| 16 | pancreatobiliary.mp. |
| 17 | exp Biliopancreatic Diversion/ or biliopancreatic.mp. |
| 18 | exp Intestine, Small/ or small bowel.mp. |
| 19 | 16 or 17 or 18 |
| 20 | limb length.mp. |
| 21 | long limb.mp |
| 22 | long tract.mp |
| 23 | length.mp |
| 24 | surgical technique.mp |
| 25 | technique.mp |
| 26 | operative technique.mp. |
| 27 | 20 or 21 or 22 or 23 or 24 or 25 or 26 |
| 28 | randomized control trial .mp. |
| 29 | controlled clinical trial.mp. or exp Controlled Clinical Trial/ |
| 30 | exp Randomized Contolled Trial/ or randomized.mp |
| 31 | exp Clinical Trial/ or trial.mp |
| 32 | 28 or 29 or 30 or 31 |
| 33 | 15 and 27 and 32 |

**Table S1d: Cochrane CENTRAL search strategy**

| **Provider/Interface** | Cochrane |
| --- | --- |
| **Database** | CENTRAL |
| **Date searched** | 01/06/2021 |
| **Search developer(s)** | Swathikan Chidambaram, Simon Erridge, Gauri Vithlani |
| **Limit to English** | Yes |

| 1 | Gastric Bypass |
| --- | --- |
| 2 | metabolic surgery |
| 3 | Bariatric Surgery |
| 4 | obesity surgery |
| 5 | weight loss surgery |
| 6 | Weight Loss |
| 7 | Metabolic Syndrome OR Diabetes Mellitus |
| 8 | Obesity |
| 9 | Surgery |
| 10 | Surgical Management |
| 11 | 9 OR 10 |
| 12 | 6 AND 11 |
| 13 | 7 AND 11 |
| 14 | 8 AND 11 |
| 15 | 1 OR 2 OR 3 OR 3 OR 5 OR 12 OR 13 OR 14 |
| 16 | pancreatobiliary |
| 17 | Biliopancreatic Diversion OR biliopancreatic |
| 18 | small intestine OR small bowel |
| 19 | 16 OR 17 OR 18 |
| 20 | limb length |
| 21 | long limb |
| 22 | long tract |
| 23 | length |
| 24 | surgical technique |
| 25 | technique |
| 26 | operative technique |
| 27 | 20 OR 21 OR 22 OR 23 OR 24 OR 25 OR 26 |
| 33 | 15 AND 27 |
