# Supplementary material for: Length of biliopancreatic limb in Roux-en-Y gastric bypass and its impact on post-operative outcomes in metabolic and obesity surgery—systematic review and meta-analysis
Source: Int J Obes (Lond). 2022 Aug 4;46(11):1983–91. doi: 10.1038/s41366-022-01186-0 (PMC9584808; doi:10.1038/s41366-022-01186-0)
Supplement: Supplementary file 7 — Supplementary Figure Legends [file 41366_2022_1186_MOESM7_ESM.docx]

Supplementary Figure 1. Forest plot of studies assessing remission or improvement in T2DM at 12 months

Supplementary Figure 2. Forest plot of studies assessing remission or improvement in T2DM at 24-60 months

Supplementary Figure 3. Forest plot of studies assessing remission or improvement in hypertension at 24-60 months

Supplementary Figure 4. Forest plot of studies assessing remission or improvement in dyslipidaemia at 24-60 months

Supplementary Figure 5. Forest plot of studies assessing incidence of complications
